# Predicting goal probabilities with improved xG models using event sequences in association football

**DOI:** 10.1371/journal.pone.0312278

**Published:** 2024-10-30

**Authors:** Ishara Bandara, Sergiy Shelyag, Sutharshan Rajasegarar, Dan Dwyer, Eun-jin Kim, Maia Angelova

**Affiliations:** 1 School of IT, Deakin University, Melbourne, Australia; 2 College of Science and Engineering, Flinders University, Adelaide, Australia; 3 Centre for Sport Research, Deakin University, Melbourne, Australia; 4 Research Centre for Fluid and Complex Systems, Coventry University, Coventry, United Kingdom; 5 Aston Digital Futures Institute, Aston University, Birmingham, United Kingdom; 6 Institute for Biophysics and Bioengineering, Bulgarian Academy of Sciences, Sofia, Bulgaria; Eötvös Loránd University, HUNGARY

## Abstract

In association football, predicting the likelihood and outcome of a shot at a goal is useful but challenging. Expected goal (xG) models can be used in a variety of ways including evaluating performance and designing offensive strategies. This study proposed a novel framework that uses the events preceding a shot, to improve the accuracy of the expected goals (xG) metric. A combination of previously explored and unexplored temporal features is utilized in the proposed framework. The new features include; “advancement factor”, and “player position column”. A random forest model was used, which performed better than published single-event-based models in the literature. Results further demonstrated a significant improvement in model performance with the inclusion of preceding event information. The proposed framework and model enable the discovery of event sequences that improve xG, which include; opportunities built up from the sides of the 18-yard box, shots attempted from in front of the goal within the opposition’s 18-yard box, and shots from successful passes to the far post.

## 1 Introduction

Kicking or heading the ball toward the opponent’s goal and penalty shots are the only ways of scoring in association football and other games like hockey and basketball. In the past literature, these sports have been categorized as invasion sports (also called “invasion games”), considering their attack-defense nature [1–3]. In contrast to the other invasion team sports, association football is low-scoring, highly unpredictable [4] and dominated by chance [5, 6]. Existing literature also shows that the goal distributions in soccer generally follow a Poisson distribution [7–9], which implies randomness in scoring, which further contributes to the unpredictability of match outcomes.

Randomness in goal scoring can be caused by the often uncontrollable factors that influence the outcome. It suggests that not all goals are solely a result of deliberate tactics, skill, or strategy; instead, some goals may occur due to unforeseen circumstances or chance [4–6]. This unpredictability can make it challenging to evaluate the team and player performance, based on the final score. To quantify the probability of a shot being a goal, a metric “Expected Goals” (xG) was introduced [10, 11]. This metric can also be used to measure the quality of the scoring opportunity. In association football, team dominance can be quantified with actual scores as well as with other performance evaluation metrics. Therefore, the use of more common performance evaluation metrics like possession, territory, shots at goal for the team and individual performance analysis has been evaluated [12]. However, there can be situations where the team that outperformed the opponent on these performance metrics, fails to win. Furthermore, team strategies may vary and there is a need to evaluate their effectiveness. Teams that follow “possession-play” may concentrate more on increasing ball possession as a winning strategy, while teams that follow “direct-play” strategies may not [13]. Moreover, assessing performance using commonly employed performance evaluation metrics such as possession and territory might be questionable, as teams employing direct-play or counter-attacking strategies may not prioritize possession and territory. The expected goal metric can serve as a good indicator of the team’s future performance in such scenarios, as the actual score or other performance evaluation metrics may not reflect the long-term value of the playing strategy [14]. Besides, xG models have also been evaluated for assessing both team and player performances [14–16], as well as for ranking teams within a league table, rather than relying solely on actual match outcomes [14].

The earliest report on expected goals in association football found that the number of goals per game increases with the number of attempted goals or shots [5]. Later, this finding was confirmed by observations that winning teams attempt more shots at goal than the losing teams [17, 18]. Many expected goals models consider shot location or the distance of the shot location from the goal [2, 11, 19–23]. During the 2006 FIFA World Cup, 32.17% of goals were scored within the goal area and 51.3% within the penalty area, while only 16.53% were scored outside the penalty area [19]. Estimation of xG scores by categorizing shots into predefined pitch zones has been a topic of investigation [24]. However, challenges can arise when categorizing shots that occur near the pitch zone boundaries. To overcome this, the use of a probabilistic clustering approach to assigning shots to pitch zones in a fuzzy manner has been explored [22]. Other factors that have been assessed for the development of expected goals models include; shooting angle [2, 11, 21], shot speed [25, 26], time in the game [11, 19], last action/event [2], and quality of the opposition [27]. The inclusion of features related to the team’s/player’s ability and physiological effects have been analyzed in recent literature by considering features like team form (i.e. team performance in recent games), team rankings, Elo rating, team’s average spend in a transfer window, and player market value [11].

Successful scoring opportunities in open play can be created via a sequence of ball movements that deceive defensive players in the sequences of play that precede a shot on goal [28]. Ignoring these sequences or related information (features characterizing the event/sequence) in the current xG models [11, 21, 22] is questionable as information that might improve the accuracy of an xG model is ignored. At their core, the state-of-the-art xG models are built upon the features of individual shots. As such, they may only provide insights about the improvement of shots only, and cannot be employed for analysis of tactical movements and buildup, which would potentially improve shot success.

Nevertheless, the use of spatiotemporal data for the development of xG models has been discussed in recent literature. Lucey et al. have examined features extracted from player and ball tracking patterns in a ten-second window preceding shots, to develop an xG model [29]. Notably, the ten-second window approach utilised in [29] might not consider full events as the physical time is not necessarily linked with the game event sequence. Furthermore, this approach may consider events that do not have a direct influence on the shot or discontinued event sequences. As an example, referee decisions for set pieces are often followed by a brief stoppage of play and subsequent restart. This interruption of play may create a discontinuity in the sequence of events.

Earlier it was suggested that the majority of goals result from three or fewer passes in a sequence with randomness playing a predominant role in football [5, 6]. This concept has been further explored in subsequent research on the randomness of goal scoring [7–9]. However, football tactics may evolve with time [30] and recent publications have considered on and off-ball movements’ contribution to creating promising opportunities during open play events [31–34]. McCarthy et al. evaluated on-ball passing sequences occurring in specific field zones that lead to or concede goal-scoring opportunities [28]. Shitansu et al. utilized on-ball player-to-player passing interactions to predict teams that create goal-scoring opportunities (shots) in event sequences [35]. Although some of these on-ball movements or passing interactions create a threat to the opposition, sometimes they may not end up with a goal-scoring opportunity. As a solution, Link et al. (2016) proposed a novel performance evaluation metric named “Dangerousity”, estimating the likelihood of a player scoring a goal while in possession of the ball at any given moment [36]. This metric calculation relies on the spatial arrangement of the player and the ball, evaluating four key components; “Zone” which evaluates the danger posed by ball carrier’s current location on the field, “Control” which evaluates the ball carrier’s ability to implement his tactical intentions, “Pressure” which evaluates the probability that the defending team can impede the actions of the ball carrier, and “Density” which evaluates chance of defending the ball after the action. However, players may not always opt to shoot at goal; rather, they might seek to generate promising opportunities for future scoring attempts. To value this, the concept of Expected Threat (xT) has emerged, which assesses the value of both chance creation and the probability of scoring at any given moment [32]. This xT metric has become prevalent in football broadcasting and analysis recently, often employed for evaluating team and individual player performance. Off-ball positioning and movements also play a crucial role in creating scoring opportunities, and to value their significance, a non-shot expected goal evaluation approach utilizing spatiotemporal player tracking data to compute off-ball scoring opportunities (OBSO) has been explored in the literature [33]. While on-ball passing sequences and off-ball runs have been considered in the development of other performance evaluation metrics, the sequence of events has not been considered in a time-series manner to improve the state-of-the-art xG models.

Solely focusing on features of the shot at goal and overlooking the features of the event sequences that lead to the shot event during the training of xG models would potentially lead to the omission of crucial information. Additionally, xG models utilising only the features extracted from shot events would fail to offer insights into event sequences that enhance the chances of scoring, if such sequences exist. Therefore, this study focuses on evaluating whether the inclusion of temporal information from preceding event sequences to the shot at goal, improves the predictive performance of xG models. In this work, the predictive performance of an Expected Goals (xG) model refers to how accurately and reliably the model predicts the probability of a goal being scored from a given shot in association football. Hence identified research questions are:

1. Do preceding events to the shot and their sequences carry useful information that improves the predictive performance of an xG model?
2. Can patterns of event sequences that improve scoring chances be identified across games?

The paper is structured as follows. Section 2, Materials and Methods, discusses methods, data, machine learning model, feature extraction, data processing, model training, and evaluation. Section 3, Results, provides cross-validation and testing results, the statistical significance analysis, model performance comparison with existing literature, model feature importance analysis, and event sequence pattern identification. Section 4, Discussion, discusses the findings, limitations, and future work. Section 5, Conclusion presents the conclusions and contributions of this work.

## 2 Materials and methods

Ethics approval for this study was obtained from the Coventry University Ethics Approval Committee (Approval Number: P174511). All procedures performed in this study were in accordance with the ethical standards of Coventry University, UK.

In this work, an “event” refers to a single specific game-related occurrence involving an action of a player or the results of these actions that could be documented. Further, in this work only events related to the ball carrier were considered for feature extraction (e.g., pass, shot at goal, carry (running with or dribbling the ball while maintaining control and possession)). Off-ball events were not considered. It could be explored in a future work. A “sequence” is a temporally ordered set of such events. “Features” may include duration of an event, angle and distance to the goal from event location.

**Fig 1:**
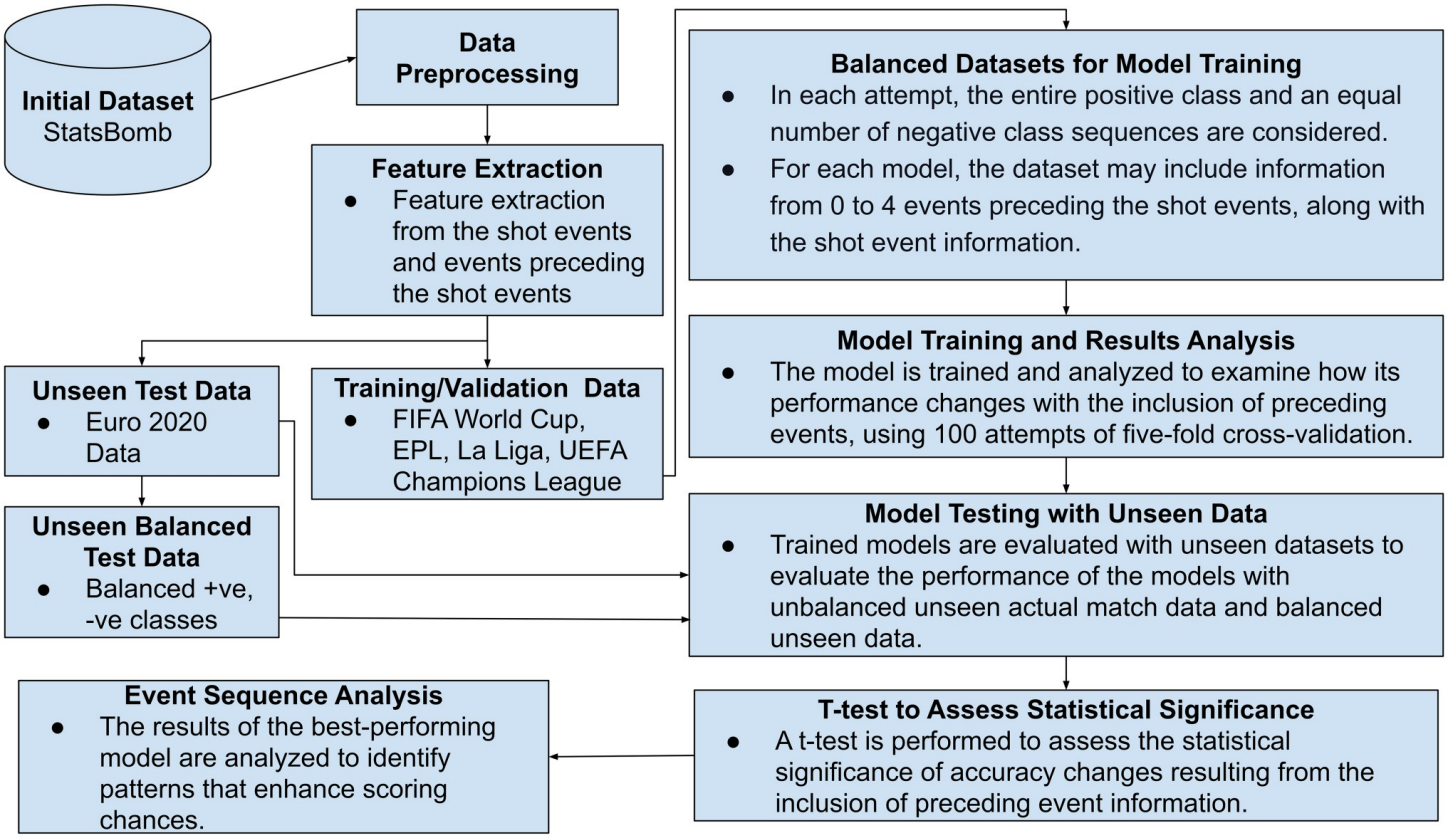
Flowchart showing the data preprocessing, feature extraction, model development, and evaluation process in summary.

This research comprises several key stages of temporal feature analysis and extraction, followed by data separation, model training, and evaluation. The evaluation process includes validation, testing using both balanced and unbalanced datasets, and a t-test to assess performance variations resulting from the inclusion of preceding events. Subsequently, the results of the best-performing model on the testing data were analyzed to identify patterns that improve scoring opportunities. Fig 1 illustrates the workflow of this study.

### 2.1 Data

A publicly available event-log data set was used (“StatsBomb Open Data”) [37]. The data set consists of matches from multiple international and top-tier European club leagues. The data includes detailed information about events that occur near the ball, player and tactical data, match information, substitutions, and information about the opposition pressure on the ball carrier.

Although the authors’ previous work has demonstrated some similarities in event distribution strategies between men’s and women’s playing strategies [38], Pappalardo et al. have identified several features where the women’s game significantly differs from the men’s game [39]. Some of these features, such as shot distance, directly impact this work’s xG model characteristics, while others, like pass distance and pass velocity, indirectly influence the features this work considers in the development of xG models. Given the statistically significant differences in these features between the two sexes, women’s game data were removed from the dataset. However, considering the proposed approach for women’s data could be evaluated as a future work. The remaining data contained the data from 682 matches in international competitions (FIFA World Cup 2018, Euro 2020) and top-tier European club competitions (England, Spain, Germany, Italy). Euro 2020 competition data consisting of data from 51 matches were separated as an unseen testing data set and the remainder was considered for training/validation.

The referee makes decisions regarding penalties and set pieces, disrupting the flow of ball movement events by players. After such decisions, there is a temporary pause in play, halting the sequence of ball movement by players. Therefore, this work did not take into account attempts that included set pieces for shots or preceding events to the shots considered. The remainder contained 12579 unsuccessful attempted goals and 1788 successful shots in open play in the training/validation split. The unseen testing split (Euro 2020) consisted of 1072 unsuccessful attempts and 121 successful attempts in open-play. The referee makes decisions regarding penalties and set pieces, disrupting the flow of ball movement events by players. After such decisions, there is a temporary pause in play, halting the sequence of ball movement by players. Therefore, this work did not take into account attempts that included set pieces for shots or preceding events to the shots considered. The remainder contained 12579 unsuccessful attempted goals and 1788 successful shots in open play in the training/validation split. The unseen testing split (Euro 2020) consisted of 1072 unsuccessful attempts and 121 successful attempts in open-play.

The original data set has a home team (“Team 1”) and an away team (“Team 2”) for each game for naming purposes. The location of each event was indicated by coordinates on a 120×80 grid, and the attacking direction is from left to right in the grid. Each event was described by its properties, such as “event duration”, “under pressure”, “body part” and “player position”. The “Event duration” property referred to how long a particular event lasted in seconds from the start of the event, the player taking control of the ball, to the end of the event with the same or different player taking control of the ball for the next named event. The “Under Pressure” property referred to whether the ball carrier was being approached by opposition defenders to disrupt the ball carrier’s actions or the team’s build-up (opposition pressure). The “Body part” property refers to the body part used by the ball carrier to perform the named action in the event (eg., “right foot” if a pass was completed by the right foot). In association football, players are formed in named positions on the field. However, they may change their positions during the game based on the situation and tactical changes. The dataset included player positions for each event under the property “player position”. The player positions property was updated to reflect new player positions if a player changed their position during the game.

### 2.2 Machine learning model

For this research, the Random Forest ensemble learning model was chosen due to its simplicity, computational efficiency, and successful application in similar problems in the existing literature [10, 21]. Although neural networks, such as bi-directional Long Short-Term Memory networks (Bi-LSTM), were initially considered, Random Forest was ultimately selected based on its performance, the dataset size, computational requirements, and its ability to provide insights into feature importance.

Random Forest (RF) is a widely used ensemble learning method for classification and regression. Generally, these ensemble models combine the predictions of multiple (sub-)models to improve the overall performance of the model. RF is an ensemble of decision trees. Each tree is trained on a random subset of the data or features. RF has become a popular machine learning approach due to its ability to handle a large number of input features, and missing values, and its easiness of usage. Further, RF is generally more robust to data without normalization compared to other machine learning algorithms (There was no noticeable performance enhancement observed with the RF models when using normalized data.). In this problem, multiple features were generated from multiple events. Therefore, RF was particularly applicable in this scenario.

### 2.3 Feature selection

In this work, a set of features (variables) that directly influence the ball-carrier’s events or event sequences and features that can be extracted in a time-series manner were considered. Based on existing literature, distance to the goal, angle to the goal, duration of the event, the shot played by foot, and opposition pressure on the ball carrier were identified as features that improve the xG accuracy and as features that could be extracted from sequences in a time-series manner. However, other common features in existing literature that cannot be extracted as time-series features, such as player/team strengths, game location, and league position of the team, were not considered. As these features are often static variables throughout the game, it was assumed that these features do not directly influence a specific event sequence’s temporal nature.

**Fig 2:**
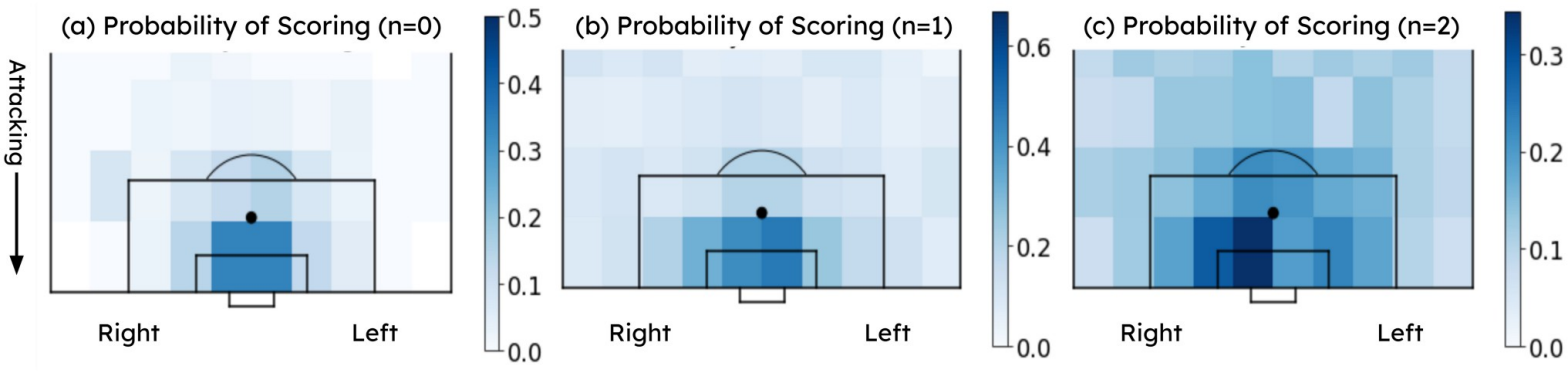
The probability that a shot will result in a goal against the shot location. Three heatmaps represent the shot locations and the probability of success from that location for events; shot event (a) *n* = 0, last events that occurred before the shot (b) *n* = 1, and second last events that occurred before the shot (c) *n* = 2 event location.

Attempted goal-scoring events (shots) and events preceding the shots (e.g., “pass”, “carry”) were evaluated for feature extraction. For naming purposes, shots were named as *n* = 0 events, and events preceding the shot were named as *n* = *i* events, where 1 ≤ *i* ≤ 5 and *i* refers to the event number preceding the shot event (e.g., *n* = 0 is the shot event, *n* = 1 is the event preceding the shot event, *n* = 2 event is the event which happened two events before the shot event). In order to analyze the spatial distribution of shots, goals, and their preceding events, a goal-scoring probability heat-map on shot locations was generated on a football field from the dataset for 0 ≤ *n* ≤ 2 (Fig 2).

By analyzing Fig 2 and from existing literature, the distance to the goal from the event location (distance to the goal) and the angle created by the event location with two goal posts of the goal (angle to the goal) was identified as two temporal features for extraction. Moreover, features such as event duration, the method of ball play (whether it’s played by foot or not), and the presence of opposition pressure on the ball carrier were identified as attributes that can be extracted in a time-series format from common features identified in existing literature as factors influencing goal-scoring probability. These temporal features extracted from the events preceding the shot and the shot itself were evaluated for their impact on goal-scoring probability. Please refer to the supporting information S1 File for this analysis.

**Fig 3:**
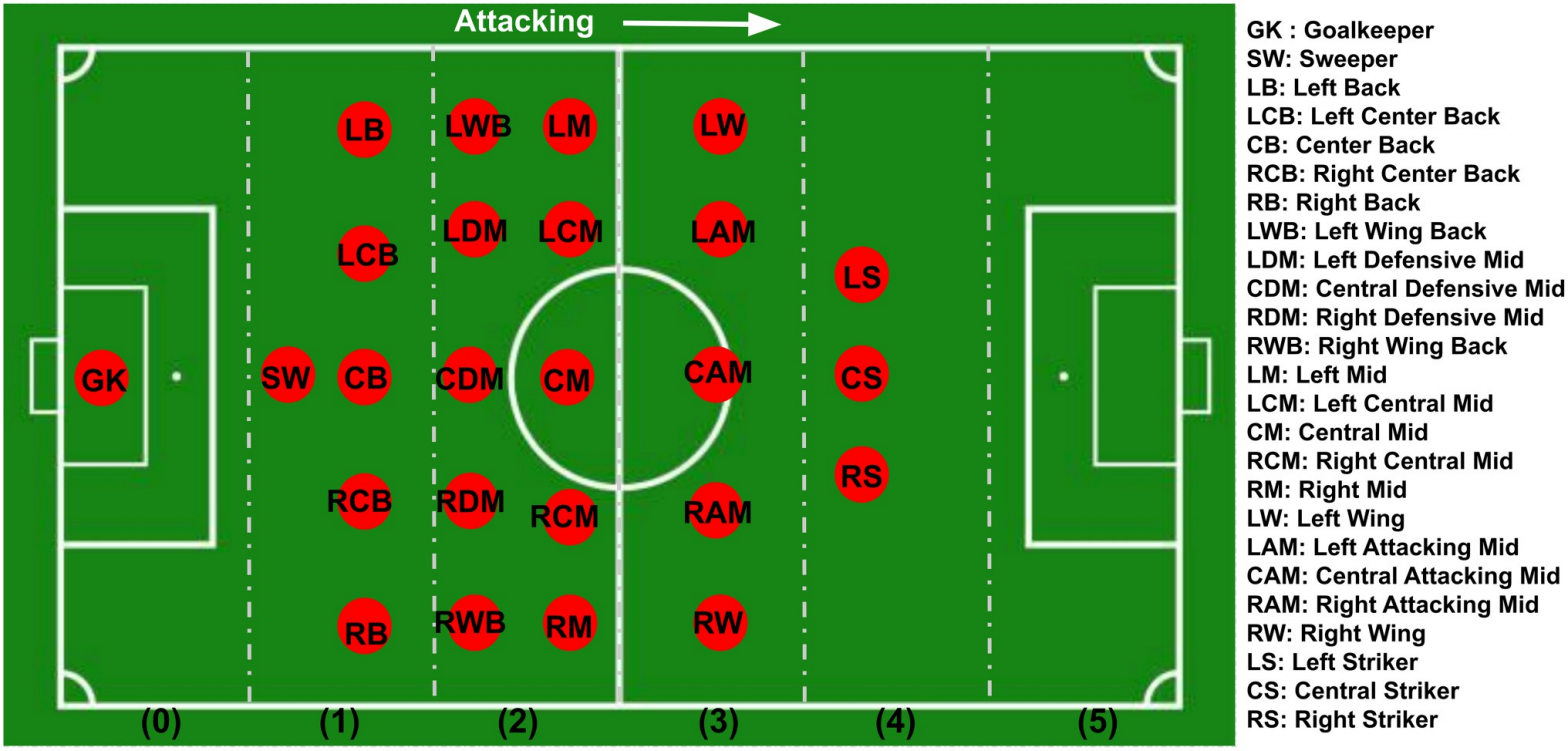
The default location of player positions with column segments that run across the field. These column segments are used to calculate an “advancement factor” that represents the relative position of a player involved in a shot at a goal.

Typically, in association football, players are assigned specific positions, as illustrated in Fig 3. By separating each line of player positions vertically, the field was segmented into six column segments (*c*) in the direction of attack (0 ≤ *c* ≤ 5). The column segments consist of the goalkeeper, defenders, defensive midfielders, attacking midfielders, and attacking forwards. In the final segment, no player position is present as it extends beyond the opposition’s defense line. These column segments are referred to as “Player Position Column” in this work. A particular player’s original player position’s column segment was considered as the “player position column” (e.g., the “player position column” of center forward (CF) is 4). However, team formation at the start of a game can be altered during the match due to substitutions and tactical adjustments. As the data set includes these changeovers, the updated team formations and positions of the players were considered for the “player position column”.

**Fig 4:**
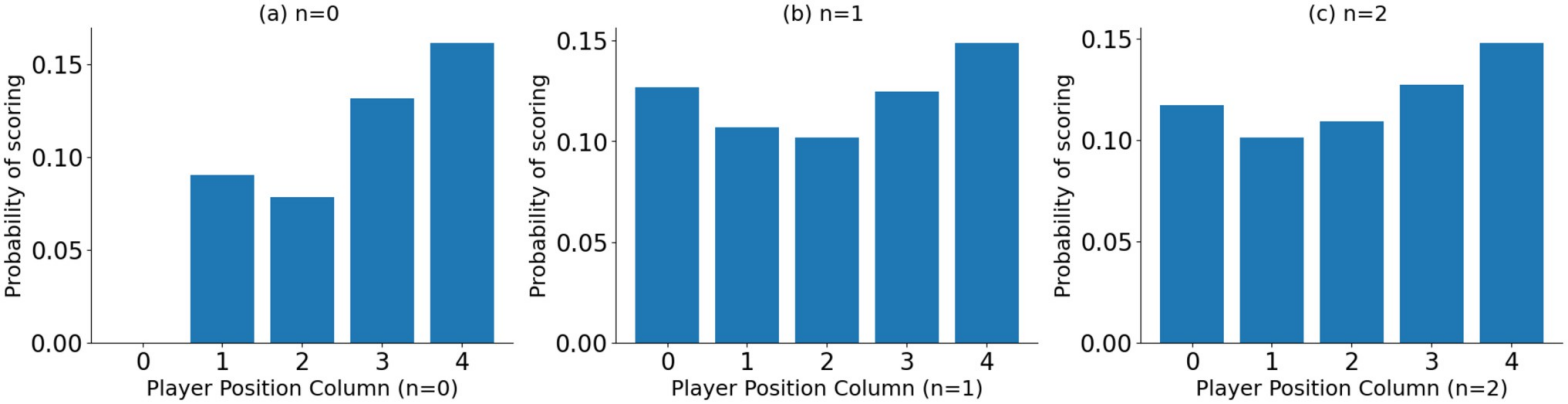
The probability of scoring against the “player position column”(i.e., column number from Fig 3). (a) shot event (*n* = 0), (b) last event before the shot (*n* = 1) and (c) two events before the shot (*n* = 2).

Fig 4 visualize the goal-scoring probability against the “player position columns”. These are independent probabilities of scoring for each “player position column”. Probabilities are calculated by dividing the number of goals for each “player position column” by the total number of shots attempted from each “player position column”. It was observed that the probability of scoring in a shot event has gradually improved with the “player position column”. This can be due to the strengths of the players, as those with greater shooting power and accuracy are generally favored for attacking positions. Similar patterns can be observed with *n* = 1 and *n* = 2 events as well. An exception was observed with “player position column” 0 (*c* = 0), which was observed to be contributed by rare goal assists by the goalkeeper. Nevertheless, since goalkeepers typically do not attempt long passes to assist in goals, the probability of success for *n* = 1 and *n* = 2 events has increased.

According to Tenga et al., the percentage of scoring from counterattacks is considerably higher (13.4%) than the general scoring attempts (8.8%) [40]. In counterattacks, teams usually regain control of the ball in the defensive third (first third) zone and then advance towards the attacking third (final third). During counterattacks initiated from the defensive third, the forwards of the attacking team may drop back to the central third or even the defensive third while midfielders may drop back to the defensive third to support defense. Similarly with tactics like “False Nine”, a forward player (typically a center forward player) drops back to central field dragging defenders with them to create open spaces in the attacking third. Moreover, with tactics like “Overlapping Fullbacks”, the defensive players (e.g., Right Back, Left Back) of the attacking team may advance forward to the attacking third overlapping wings (e.g., RW, LW) while mid-fielders move closer to the goal area improving the numerical superiority of attacking team’s players close to the opposition goal. In order to capture and quantify these advancements or the dropping back of players in each event sequence, the “advancement factor” was introduced in this work.

**Fig 5:**
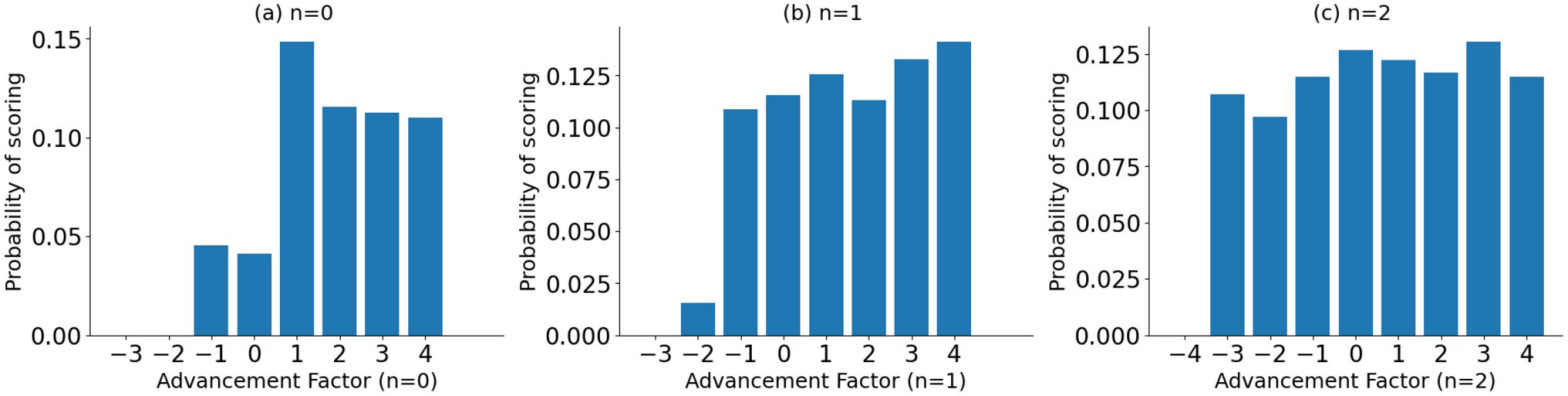
The probability of scoring against the “advancement factor”. (a) shot event (*n* = 0), (b) last event before the shot (*n* = 1) and (c) two events before the shot (*n* = 2).

The “advancement factor” was computed as the difference between the segment number of the event’s location column segment and the “player position column” of the ball carrier. In the dataset, event locations are denoted on a 120 × 80 grid. After segmenting the field into six column segments, as shown in Fig 3, each segment represents a 20 × 80 column, which was used to determine the event’s location column segment. As an example, if a Right Wing (RW) player takes a shot from inside the opposition’s 18-yard box, the “advancement factor” of that particular shot event is 2. The “player position column” number for RW is 3 (*c* = 3), and the event location column segment number for this particular event is 5 (*c* = 5). Therefore, the player has advanced 2 column segments from his original position to take this particular shot. If the RW player of the same team goes back to collect and perform a pass from column segment 1, the “advancement factor” of that particular event is -2. Fig 5 shows the scoring probability against the “advancement factor” for the shot events and *n* = 1, *n* = 2 preceding events. These are independent probabilities of scoring for each “advancement factor” calculated by dividing the number of goals scored with each “advancement factor” by the number of shots attempted from each “advancement factor”. According to these plots, the probability of scoring improves with the “advancement factor” for the shot (*n* = 0). However, the highest probability can be observed for “advancement factor” 1. This could be due to strikers (such as LS, CS, RS) scoring from close range to the goal, as they typically excel in shooting power and accuracy. For *n* = 1 and *n* = 2 events, gradual improvement in scoring probability can be observed with the “advancement factor” where negative advancements have low scoring probabilities. This may indicate that players advancing in the build-up may improve the chance of scoring. The highest probability of scoring for *n* = 1 events is achieved when defensive players of the attacking team (e.g., RB, LB) have advanced 4 columns (e.g., tactics like overlapping fullbacks). In such instances, the numerical advantage of attacking team players near the opposition goal is enhanced, leading to an improvement in their ability to win the ball and, consequently, an increased likelihood of scoring.

After evaluating various features, seven of the temporal features described above were selected for temporal feature extraction based on the patterns observed. These were all extracted from the shot event and multiple events preceding the shot event (see Table 1.).

**Table 1:** The features that were evaluated for inclusion in the xG model. The values of all features were determined for shot events and the events preceding the shot.

*Feature (abbreviation)*	*Type*	*Value Range*
Duration of the event (du)	Numerical	0–5
Angle to the goal (a)	Numerical	0–180
Distance to the goal (di)	Numerical	0–120
Advancement factor (ad)	Numerical	(-5)-5
Player position column (pp)	Numerical	1–5
Played by foot or not (if)	Binary	0,1
Is the player being pressured by the opposition (ip)	Binary	0,1

### 2.4 Data processing

**Fig 6:**
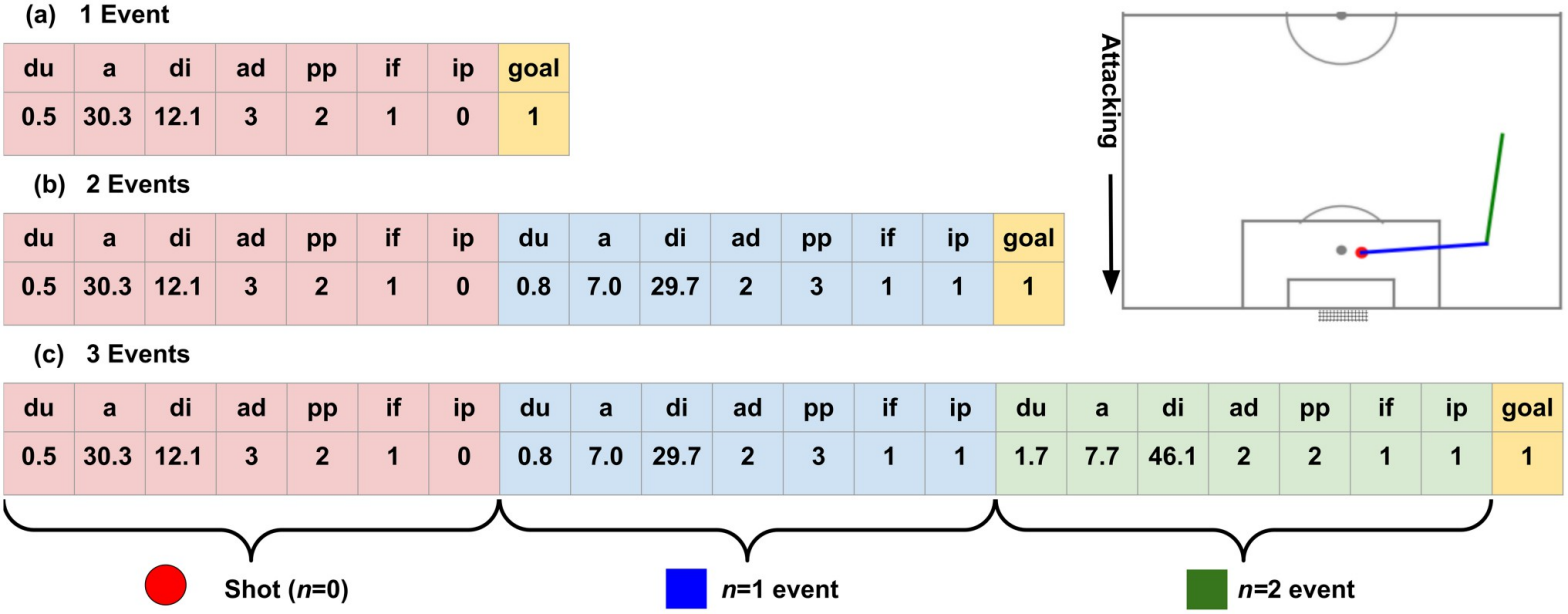
Data input formats for models. (a) 1 event (single event model), (b) 2 events, and, (c) 3 events for a sequence example shown in the top right.

Both shot event only models (the ones trained only with shot event information) and multiple event (multi-event) models were evaluated. Table 2 and Fig 6 describe the approaches and the events included for each of single-event or multi-event models.

**Table 2:** Approaches and included features.

*Approach*	*Events included*	*Total Features for RF Input*
1 event	*n* = 0 (shot) event	1 × 7 = 7 features
2 events	*n* = 0, *n* = 1 events	2 × 7 = 14 features
3 events	0 ≤ *n* ≤ 2 events	3 × 7 = 21 features
4 events	0 ≤ *n* ≤ 3 events	4 × 7 = 28 features
5 events	0 ≤ *n* ≤ 4 events	5 × 7 = 35 features

The event sequences contain both events where the team that completed the shot event is in possession, and the opposition is in possession. In order to differentiate these two types of events, the event duration value was considered as a positive value when “Team 1” was in possession and as a negative value when “Team 2” was in possession. The remaining features were extracted based on the location of the opposition goal relative to the team in possession of the ball.

### 2.5 Model training

RF regression models were trained to produce probabilistic classification task predictions. The fine-tuned RF model consists of 100 estimators and a random state of 42. No depth limitation was used for “Maximum Depth” of the model. “Minimum Samples Leaf” and “Minimum Samples Split” were set to 1 and 2 respectively.

One hundred (100) rounds of model training and validation with five-fold cross-validation were performed. Given the significant imbalance in the data between positive class (goals) and negative class (unsuccessful shots), balancing pre-processed data sets with random under-sampling was performed with each round of five-fold cross-validation to balance the training and validation data in each round. Random under-sampling was performed independently in each round of model training and validation to minimize information loss and other adverse effects associated with sampling, as reported in the literature [41–43]. Prior to sampling, the generated pre-possessed unbalanced data sets containing temporal sequences were shuffled to randomize the order of data samples. All the goal sequences and an equal number of randomly picked unsuccessful scoring attempt sequences were picked for a pre-processed balanced data set which was used for model training and validation.

### 2.6 Evaluation

In addition to 100 rounds of five-fold cross-validation, each model from the 100 rounds of cross-validation (500 models) was further tested with both balanced and unbalanced testing data. The unbalanced testing dataset consisted of all attempted scoring sequences from the unseen Euro 2020 dataset. For the balanced testing data, preprocessed sequences from the Euro 2020 dataset were randomly under-sampled in each testing round to ensure an equal number of goal and unsuccessful sequences.

The Brier score, which assesses the accuracy of probabilistic predictions and provides insights into the calibration of predictions with actual outcomes, was considered as an evaluation metric. It ranges from 0 to 1, where 0 indicates perfect predictions and 1 indicates completely inaccurate predictions. However, it is sensitive to class imbalance. Therefore, “Receiver Operating Characteristic—Area Under the Curve” (ROC-AUC), a metric that remains robust even in the presence of significant class imbalance, was also considered, given that the original and test datasets exhibit substantial imbalances between positive and negative classes. ROC-AUC ranges from 0 to 1, where 1 indicates a perfect model prediction. ROC-AUC distributions from 500 evaluations for each approach underwent further analysis using a t-test to determine whether improvement in mean ROC-AUC values due to the inclusion of preceding event information were significant.

## 3 Results

### 3.1 Cross validation results

**Fig 7:**
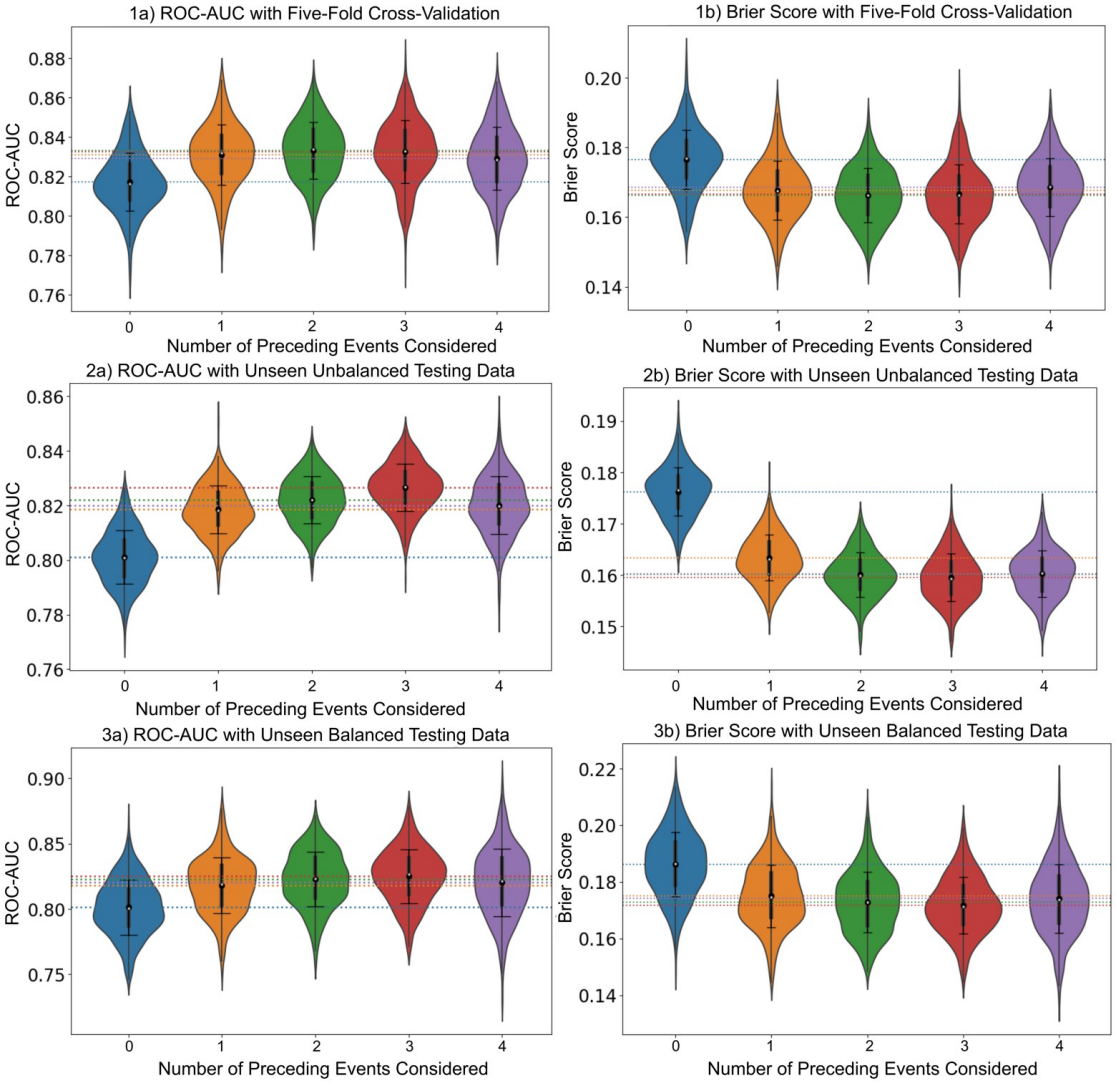
Violin plots of ROC-AUC and brier score. Violin plots displaying 500 evaluations of validations, with 100 attempts for (1a, 1b) five-fold cross-validation, (2a, 2b) testing with unseen unbalanced data, and (3a, 3b) testing with unseen balanced data.

From Fig 7(1a) and 7(1b), it was observed that ROC-AUC values improved and brier scores values decreased gradually up to inclusion of three events (shot event and two preceding events to the shot event) indicating an improvement in model performance. This was followed by a slight drop in model performance when four events were included and a significant drop in model performance when five events were included in the input sequence. The highest mean validation ROC-AUC of 0.833 and lowest mean brier score of 0.166 were achieved with models trained with information from three events (shot event and two preceding events).

### 3.2 Unseen testing data results

Trained models were further tested with unseen Euro 2020 data. Models were evaluated with unseen unbalanced original Euro 2020 data and unseen balanced Euro 2020 data.

Results with both test data sets produced similar results. The ROC-AUC increased with the inclusion of preceding event information. Performance improved up to the inclusion of information on the three preceding events along with the shot event (4 events in total). The Brier score decreased with the inclusion of the preceding events. Fig 7(2a), 7(2b), 7(3a) and 7(3b) show violin plots of how the ROC-AUC and Brier score changed with the inclusion of preceding event information with unbalanced unseen testing data and with balanced unseen testing data respectively.

### 3.3 Performance fluctuation assessment

The t-test and Mann-Whitney U test (Mann-Whitney-Wilcoxon) were considered to evaluate the statistical significance of performance improvement when information from events preceding the shot was included as time-series data. Uni-variate normality tests such as Shapiro-Wilk test and Anderson-Darling test were conducted to assess the normality of the results distributions. Once it was confirmed that the distributions were almost normal, the t-test results were used to evaluate the statistical significance of performance improvement in multi-event models.

For the t-test, only ROC-AUC distributions are presented here as Brier score distributions demonstrated similar results. As the null hypothesis it was assumed that there is no significant difference for performance evaluation distributions between single-event models and multi-event models. Further, including information on the events preceding the shot event improves performance was considered as the alternate hypothesis for the t-test.

Table 3 shows the results of the t-test. ROC-AUC distributions with each inclusion of preceding event information (*n* events where *n* > 1) were compared against ROC-AUC distribution with shot event information only. In these tests, single-event model ROC-AUC distribution was considered as the first distribution, and multi-events model ROC-AUC distributions were considered as the second distribution for comparison. Therefore, negative *t* values suggest that including preceding event information has improved the mean ROC-AUC in multiple-event models. Very low p-values of approximately zero (< 0.05) prove a statistically significant difference between the ROC-AUC distribution of single-event models vs multi-event models. This supports the evidence that the null hypothesis can be rejected and that the alternate hypothesis should be accepted.

**Table 3:** t-test results of comparison between single-event models and multi-event models.

*Data*	*Models Compared*							
	*1 vs 2 events*		*1 vs 3 events*		*1 vs 4 events*		*1 vs 5 events*	
	*t value*	*p-value*	*t value*	*p-value*	*t value*	*p-value*	*t value*	*p-value*
Val.^1^	−14.5	1.9 × 10^−43^	−17.5	3.8 × 10^−60^	−15.8	1.5 × 10^−50^	−12.3	2.3 × 10^−32^
Unbal. Test^2^	−29.7	3.1 × 10^−139^	−35.8	2.0 × 10^−181^	−43.6	4.3 × 10^−233^	−29.5	3.1 × 10^−138^
Bal. Test^3^	−12.7	3.9 × 10^−34^	−16.3	4.4 × 10^−53^	−18.1	5.3 × 10^−64^	−12.8	7.7 × 10^−35^

^1^ ROC-AUC values obtained from 500 evaluations with validation data using 100 rounds of five-fold cross-validation

^2^ ROC-AUC values obtained from 500 evaluations with unseen unbalanced testing data

^3^ ROC-AUC values obtained from 500 evaluations with unseen balanced testing data

### 3.4 Comparison with past work

The proposed novel models have demonstrated better results in comparison to those in the existing literature. Table 4 compares the performance of this work’s models with recent publications. This work’s models have achieved higher ROC-AUC values compared to those models where the shot event only has been assessed.

**Table 4:** Comparison with existing literature (previously published models were trained on shot event only).

*Research*	*Model*	*ROC-AUC*	*Evaluation Criteria*
Eggels (2016) [10]	Random Forest	0.775	Average cross-validation performance with event-log+player-tracking data from 5020 professional games
	Decision Trees	0.777	
	Logistic Regression	0.785	
	ADA Boost	0.692	
Anzer and Bauer (2021) [21]	Random Forest	0.794	Unseen 20% a test data split from an event-log data set consist of 105,207 shots in the German Bundesliga
	GBM	0.822	
	Logistic Regression	0.807	
	ADA Boost	0.817	
Haaren (2021) [22]	Boosting Machine	0.793	Unseen event-log test data consist of 38,737 shots in European football leagues
Mead (2023) [11]	XGBoost	0.800	30% test data split from WYSCOUT data set [44, 45]
The present work			Average performance from 100 rounds of five-fold cross-validation + average performance with unseen unbalanced and balanced testing data from 500 models developed
Validation Data	**Random Forest (3 events)** ^1^	**0.833**	
Unbalanced testing	**Random Forest (4 events)** ^2^	**0.827**	
Balanced testing	**Random Forest (4 events)** ^3^	**0.826**	

^1^ The highest mean ROC-AUC value with validation data was obtained when shot event information and two preceding events information (3 events) were included (0 ≤ *n* ≤ 2 events)

^2^ The highest mean ROC-AUC value with unseen unbalanced testing data was obtained when shot event information and three preceding events information (4 events) were included (0 ≤ *n* ≤ 3 events)

^3^ The highest mean ROC-AUC value with unseen balanced testing data was obtained when shot event information and three preceding events information (4 events) were included (0 ≤ *n* ≤ 3 events)

### 3.5 Feature importance analysis

**Fig 8:**
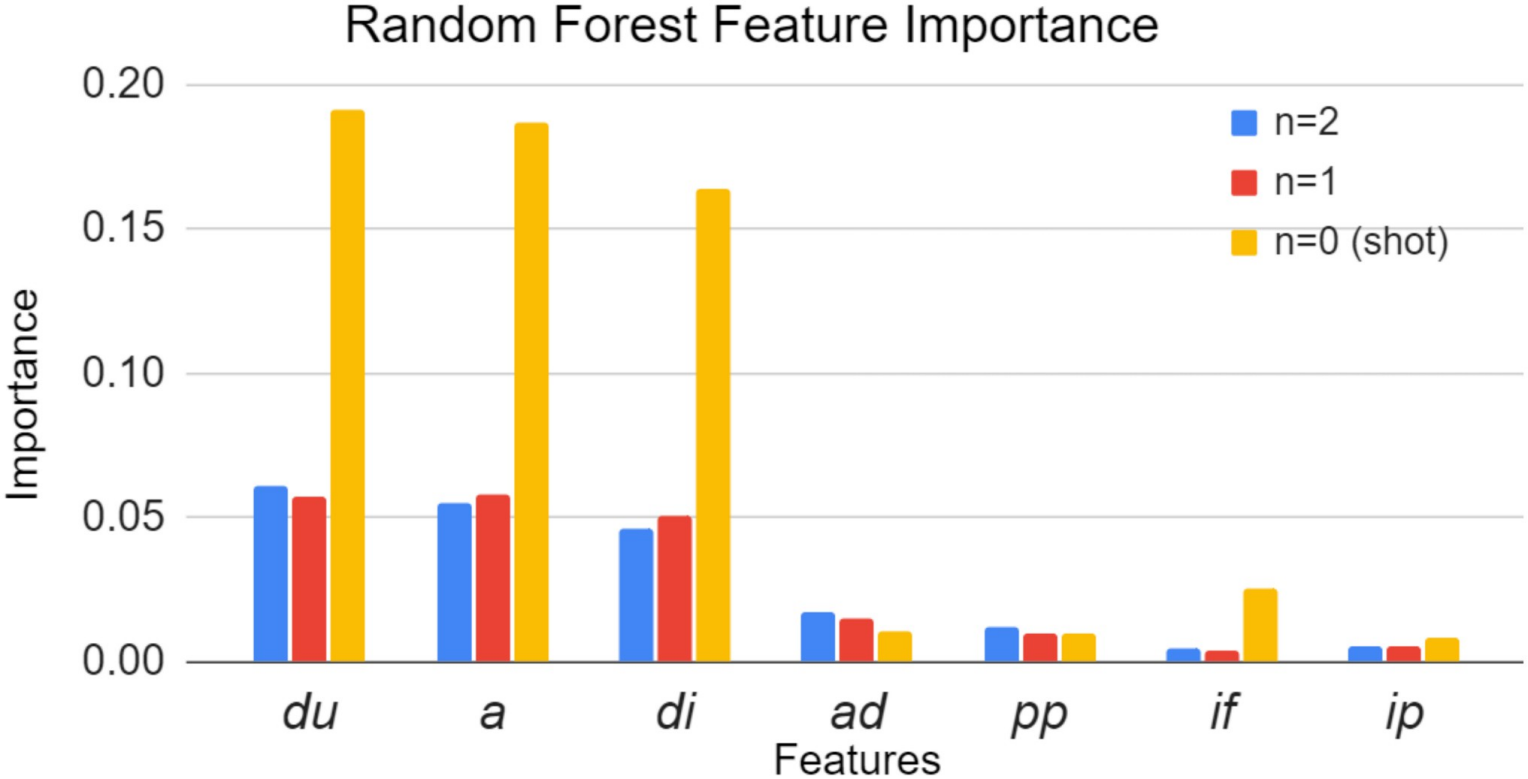
The average feature importance by the models trained with data from three events.

The best-performed models with five-fold cross-validation (models trained with three events) were further evaluated for their average feature importance. Fig 8 indicates the average feature importance from the highest mean validation ROC-AUC achieved approach (3 events).

From the feature importance plot, it can be observed that although more importance has been given to the shot event features, considerable importance has also been given to the events preceding the shot event. The best-performing model highlighted distance to goal, angle to goal, and duration of the event as critical for all three events. The novel features introduced in this study, the “advancement factor” and “player position column”, were given higher importance for events preceding the shot compared to features like whether the ball carrier was pressured (*ip*) and whether the action was played by foot (*if*), which have been commonly used in existing literature.

### 3.6 Sequence patterns that improve the success probability

To evaluate the significance of event sequences and determine if the xG model relies on meaningful patterns, an experiment was conducted by developing a similar model using the proposed approach on a randomly generated event sequence dataset. This dataset included shot events and two preceding events (similar to the best-performing model), with value ranges specified in Table 1. The resulting model achieved accuracy and AUC-ROC close to 0.5, indicating that the xG model depends on meaningful patterns in event sequence data, as the random event sequence data failed to provide predictive power. This challenges the notion that goal scoring is highly influenced by randomness or luck [4], suggesting that preceding event sequence patterns significantly impact the chances of scoring. However, goals purely due to luck can occur in football (e.g., a goal kick with no intention of goal scoring resulting in a goal) and typically have very low xG values, reflecting their rarity based on historical data. These goals do not reflect an intentional playing strategy or a performance that can be coached. This analysis is, in part, intended to assist in the development of coachable playing strategies that can help teams.

After identifying the significance of event sequences for goal scoring, the xG values obtained from the trained models for successful and unsuccessful scoring attempt sequences in the unseen test dataset were analyzed to detect sequence patterns and areas that enhance the chance of scoring. The model trained with three events (three-event model) achieved the best average ROC-AUC and Brier score values. The highest ROC-AUC value achieved by a three-event model with an unseen unbalanced testing data set was 0.844 (validation AUC-ROC of this model was 0.872). This model was used to obtain xG values for successful and unsuccessful goal-scoring attempt sequences unseen unbalanced testing data from Euro 2020. The xG values were then analyzed to identify sequence patterns that enhance scoring chances. Obtained xG values with the unseen unbalanced testing data set (AUC-ROC 0.844) were clustered using the K-means clustering algorithm based on the predicted xG value. The optimal number of clusters was decided by the silhouette score and the cluster distribution. The xG values and corresponding sequences were separated into 3 clusters. Mean xG values were calculated for each cluster. The highest mean xG (high xG) cluster received a mean xG score of 0.742, while the lowest mean xG cluster(low xG) and the other cluster (mid xG) received mean xG scores of 0.120 and 0.388, respectively.

**Fig 9:**
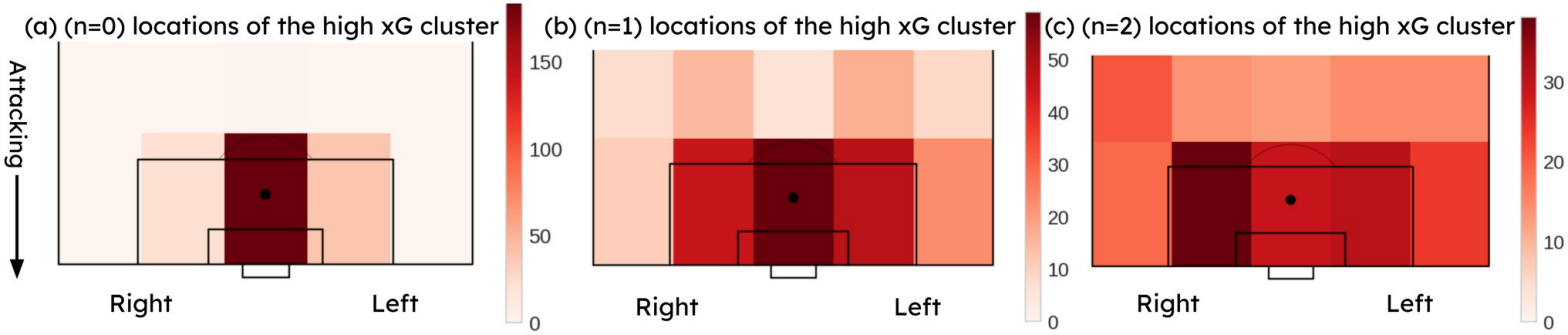
(a) *n* = 0, (b) *n* = 1, (c) *n* = 2 event locations of the high xG cluster.

Fig 9 indicates the *n* = 0, *n* = 1, *n* = 2 event locations of the high xG cluster. Fig 9 shows that most of the high xG chances were created from passes from the two sides of the goal. Fewer high xG opportunities have been created from *n* = 2, *n* = 1 events in the central zone outside the 18-yard box.

**Fig 10:**
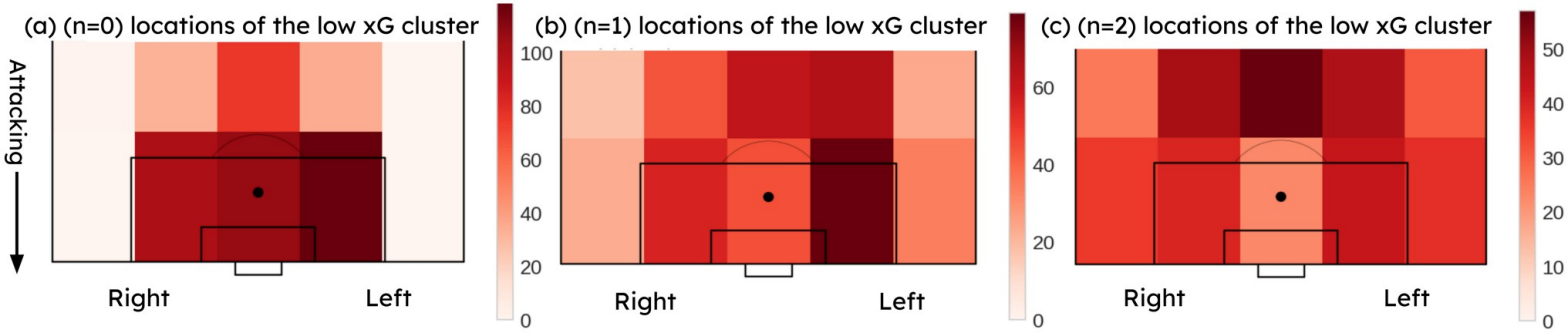
(a) *n* = 0, (b) *n* = 1, (c) *n* = 2 event locations of the low xG cluster.

Fig 10 shows the *n* = 0, *n* = 1, *n* = 2 event locations of the low xG cluster. It can be observed that most of the shots have been attempted from the sides of the goal and somewhat further away from the goal compared to the attempts in the high xG cluster. A considerable amount of attempts were built up from the central zone (*n* = 1, *n* = 2 events) outside the 18-yard box.

**Fig 11:**
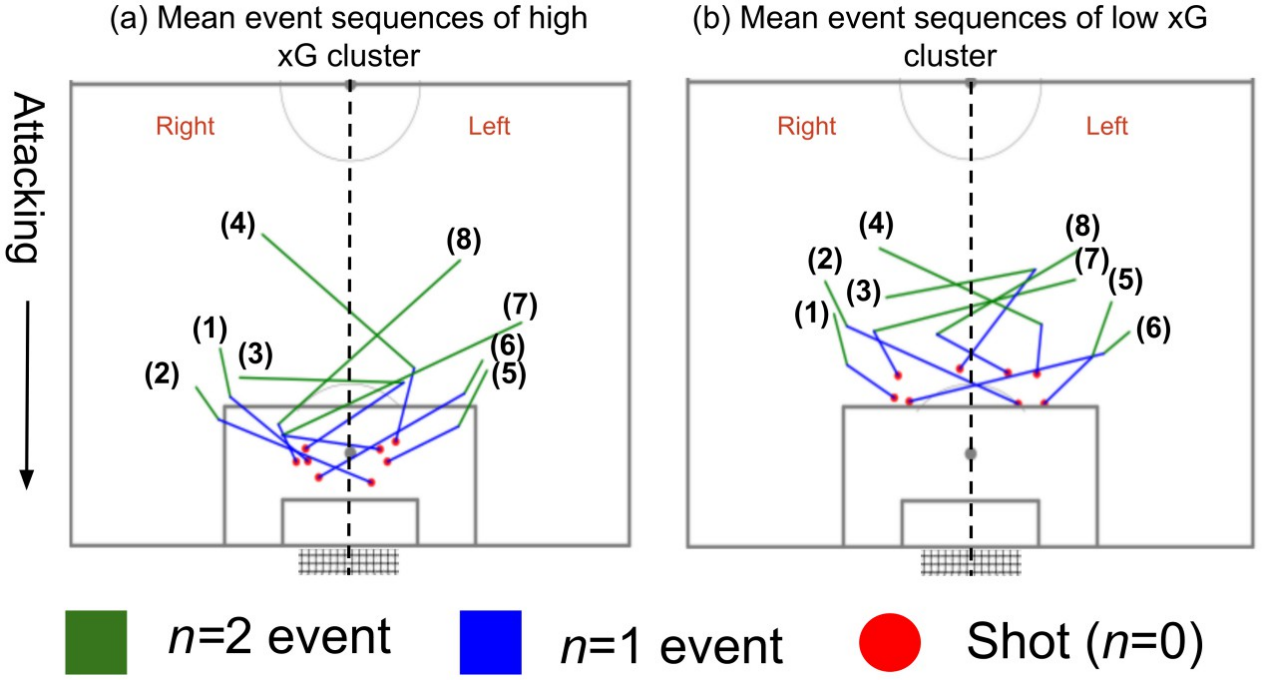
Mean event sequence positions in right and left vertical halves for *n* = 0, *n* = 1, *n* = 2 events. (a) high xG cluster; (b) low xG cluster.

Next, event sequences from the xG clusters were used to identify *mean event sequence patterns*. The field was divided into two halves vertically (left and right half) towards the attacking direction, connecting the center spot and penalty spot. Mean xG values were identified for eight possible sequences and provided in Table 5. Additionally, mean position values were calculated for *n* = 0, *n* = 1, and *n* = 2 events for each sequence in the three xG clusters as *mean event sequence* positions. Mean event sequences of low xG and high xG clusters are shown in Fig 11.

**Table 5:** Event sequences and their mean xG. The mean value for xG is denoted as *μ* xG.

*No*	*Sequence*	High xG		Mid xG		Low xG		*μ xG*
		*Nos*	*μ xG*	*Nos*	*μ xG*	*Nos*	*μ xG*	
(1)	right, right, right	69	0.77	109	0.38	152	0.12	0.34
(2)	right, right, left	37	0.76	41	0.43	46	0.16	0.43
(3)	right, left, right	5	0.7	09	0.37	4	0.06	0.39
(4)	right, left, left	13	0.71	24	0.36	44	0.12	0.29
(5)	left, left, left	56	0.73	85	0.39	139	0.11	0.32
(6)	left, left, right	35	0.73	38	0.38	21	0.14	0.46
(7)	left, right, left	7	0.75	10	0.41	7	0.10	0.42
(8)	left, right, right	17	0.74	18	0.38	56	0.10	0.27

By analyzing Table 5 it was identified that sequence (2) “right, right, left” (*μ* x*G* = 0.43) and sequence (6) “left, left, right” (*μ* x*G* = 0.46) have been more effective than the other sequences based on their mean xG (*μ* xG) values. Sequence (6) can be considered as the mirror sequence of (2). Nearly 1/3 attempts of these two sequences have been classified as high xG attempts by the model.

Fig 11 shows that sequences (2) and (6) have been built up from a shallow angle to the goal, and a shot has been attempted from the opposite vertical half (left, right) close to the far post from the location of the *n* = 1 event. The sequences revealed that the angle formed between the location of the *n* = 1 event, the midpoint of the goal, and the location of the *n* = 0 event was higher in these two sequences compared to the others. This could pose a challenge for goalkeepers, as they would need to cover a larger angle or ground, potentially compromising their stability and positioning for making a save. Additionally, if the goalkeeper is positioned close to the near post to save a potential shot from *n* = 1 event location, pressures or commits to the *n* = 1 event, it could potentially result in an easy open-goal “tap-in” opportunity for the player positioned at the far post. “Tap-in” opportunities refer to a situation where a player has a straightforward opportunity to score a goal by simply tapping the ball into the net as the goal is unguarded or the goalkeeper is out of position. In both of these sequences, the *n* = 1 event is a pass across the goal to the far post from right to left or left to right vertical halves. The next highest xG sequences, sequence (3) (*μ* x*G* = 0.39) and sequence (7) (*μ* x*G* = 0.42), also suggest a pass to the far post for the *n* = 1 event. Therefore, it can be concluded that scoring attempts from successful passes to the far post are more effective than attempts from successful passes to the near post. However, it can be relatively tricky to successfully pass to a teammate at the far post as the pass has to go past multiple opposition players to reach the teammate at the far post. Therefore, these sequences have been attempted fewer times than sequence (1) and sequence (5), where no pass is attempted towards the far post.

**Fig 12:**
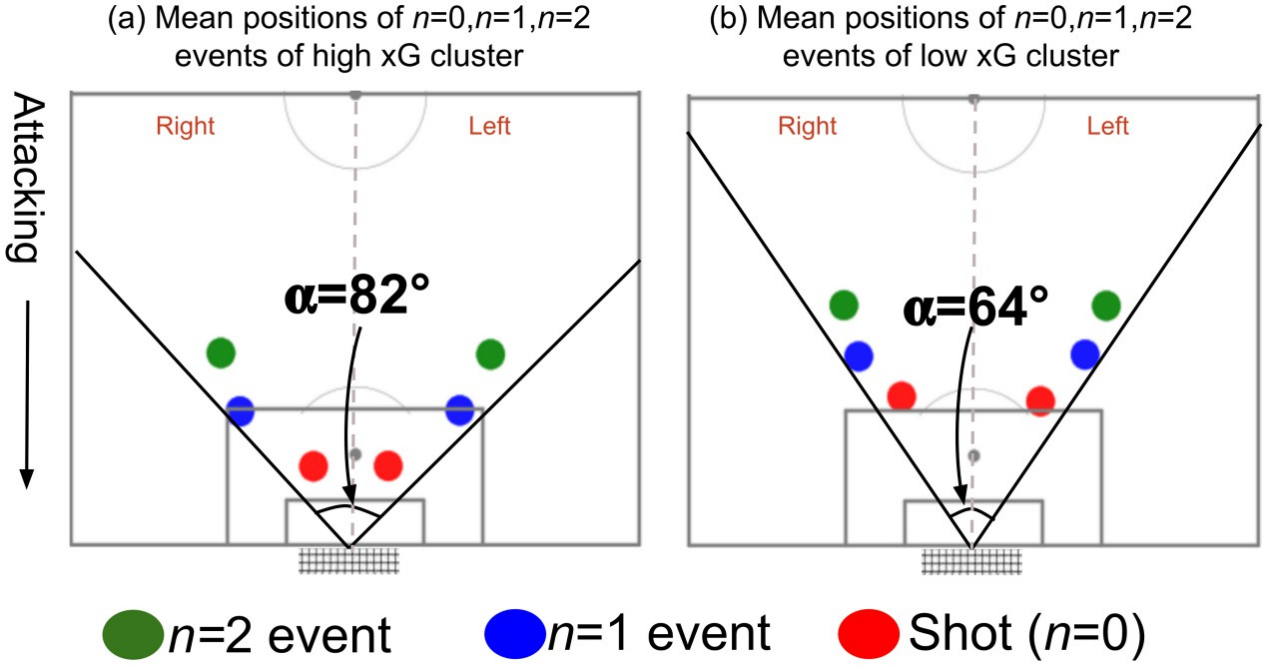
Mean positions of *n* = 0, *n* = 1, *n* = 2 events in the right and left vertical halves (a) high xG cluster; and (b) low xG cluster.

For further analysis, the mean positions of *n* = 2, *n* = 1, and *n* = 0 events in the right and left halves were computed for both the high xG cluster and the low xG cluster (Fig 12). By analyzing these mean positions (Fig 12), one can see that the maximum angle (*α*) created by these with the mid-point of the goal line (assuming that the opposition goalkeeper’s point of view is placed there) is considerably higher in the high xG cluster (82 degrees) than in the low xG cluster (64 degrees). Also, the mean positions of the preceding events in the high xG cluster are closer to the goal compared to those of the low xG cluster. This further suggests that more opportunities in higher xG clusters are being created by passes from the sides of the goal and closer to the goal. Given that the final events are happening in close proximity to the goal and widely spaced apart, the opposition goalkeeper has to cover a greater ground in a shorter reaction time, making these sequences harder to save. Additionally, when more space on the sides is utilized in *n* = 1, *n* = 2 events, opposition defenders can be drawn towards the sides of the goal. Eventually, this can lead to open space in front of the goal.

A common sequence of events that improves the probability of scoring was observed from Figs 2, 9–12. Fig 2 suggested that the highest probability of scoring was associated with shots built up from the sides of the goal within the 18-yard box, as obtained from the actual scores. Figs 9 and 10 reveal the same areas based on xG score results. Figs 11 and 12 compare the probable mean event sequences of the high and low xG clusters, which suggests that the sequences of high xG cluster are built up from the sides of the goal within the 18-yard box, whereas the sequences of low xG cluster are built up towards the central zone outside the 18-yard box. The majority of shot events, along with the two events immediately preceding the shot in the high xG cluster, provide additional evidence that most high xG opportunities are created from short passes near the goal within the 18-yard box. In all these figures, a higher probability can be observed for shots attempted in proximity to the goal with a greater angle to the goal. Fig 11 and Table 5 reveal that chances created at the far post through passes across the goal have been more effective than those created at the near post. Therefore, it can be concluded that the probability of scoring can be improved by:

1. Attempting to score from close proximity to the goal inside the opposition’s 18-yard box with a shorter distance to the goal and greater angle to the goal;
2. Attempting to create more chances from two sides of the goal inside the 18-yard box instead of the central zone;
3. Attempting to create more chances at far post instead of near post.

Hence, it is reasonable to conclude that employing passes and cutbacks from the sides of the 18-yard box to create scoring opportunities is a more effective approach than attempting to create chances from the central zone or from further away with long passes.

## 4 Discussion

This study presented a novel contribution to the widely used expected goals models in association football by evaluating whether information from events preceding the shot event, improves the performance of expected goal models. Results with random forest models demonstrate that while the main contribution to the performance of the expected goals model is created by the features of the shot event, the inclusion of temporal information from events preceding the shot and their sequences improved the accuracy of the model. The highest validation performance was shown with the inclusion of two preceding event information in addition to the shot event information. The best performance with unseen test data was shown with the inclusion of three preceding events information. A slight drop in performance was observed when four preceding event information were included. Nevertheless, models showed a better performance even with the inclusion of four preceding event information than with only the shot event. Multi-event models trained with information from the shot event and events preceding the shot event achieved higher average ROC-AUC values than single-event (shot-based) models available in the literature. Recently published top-performing models, Anzer and Beuer (2021) [21], as well as Mead (2023) [11], attained ROC-AUC scores of 0.822 and 0.800, respectively, during validation with 20% and 30% test splits. The best-performing models proposed in this study achieved an average AUC-ROC of 0.833 with 100 rounds of five-fold cross-validation and 0.826 with a separate, unseen test dataset, underscoring the importance of the proposed approach.

Using the best-performing model, this study further evaluated the event sequence patterns that improve the chances of scoring, as opposed to the existing xG models that suggest improvements to shot events only. Results revealed that attempts created from the sides of the goal in the opposition’s 18-yard box followed by shot attempts from in front of the goal improved the chances of scoring. Further, shots at the goal preceding successful passes to the far post improved the chances of scoring compared to shots at the goal from successful passes to the near post.

A relatively small data set was used in this work and was implemented only for association football games. Nevertheless, the same approach can be implemented for other similar invasion sports like hockey, basketball, and handball. This work only considered ball-carrier event sequences and not information about the activity of players off the ball, due to the unavailability of that data. Moreover, focusing solely on the ball carrier’s relative position in the “advancement factor” might not consistently portray the overall team’s relative positioning and progression on some occasions. While existing literature has discussed the significance of opposition goalkeepers’ positioning as a factor influencing the probability of shot success [21, 29], it was not considered due to dataset limitations, despite being identified as a feature that could potentially be temporally extracted (Original dataset does not contain goalkeeper position for non-shot events). These off-ball movements and opposition positions could be considered for a future analysis.

In association football, the ratio of shots to goals is highly imbalanced. To balance the training and testing data, a random under-sampling approach was used. To minimize information loss and other effects of resampling, this study conducted a comprehensive evaluation of models using 100 rounds of five-fold cross-validation with random under-sampling in each attempt. However, despite these measures, the reliance on under-sampling remains a limitation of this work due to potential information loss.

It should also be noted that this approach can only be applied to shots in open play where there is no discontinuation in event sequences with referee interaction. Shots taken directly from set-pieces including penalties, cannot be evaluated with the proposed approach, as there is discontinuation in play due to referee interaction. Therefore, the models developed in this work may not be ideal for generating the total expected goal score for an entire association football game.

The original dataset contained matches from after 2004. However, game-play strategies evolve [30] and may change over time. Since this work uses a simple random forest model, which provides information about feature importance, new models trained with the same approach using data with evolved tactics could help investigate how strategies in association football evolve. Changes in the relative importance of features and sequences that enhance scoring chances as revealed by models trained with evolved tactics, would provide insights into how the game has evolved.

## 5 Conclusion

In conclusion, this study presented a novel expected goal model by evaluating the impact of preceding events and their sequences on xG model performance. Integrating information from events preceding the shot event as temporal features enhanced the performance of the xG model. This emphasized the significance of considering the information provided by preceding actions in understanding goal-scoring opportunities.

Furthermore, the analysis of xG visualizations and model features, revealed that passes originating from the sides of the goal within the 18-yard box in preceding events tend to create higher scoring chances compared to shots built up from passes originating from the central zone outside the box. Additionally, passes to the far post followed by a shot from close range improve success probability than opportunities created from passes to the near post.

In summary, the likelihood of scoring a goal is not solely attributed to the final shot event but is affected by the sequence of events leading up to the shot. These events, although varied in their contributions, collectively play a role in creating a successful scoring opportunity. Moreover, the identification of common sequences that enhance scoring chances highlights the impact of strategic build-up tactics.

Findings from this work challenge the notion that football results, in general, are highly susceptible to random influences or luck [4], suggesting the importance of event sequences which involves deliberate execution of plans, strategic maneuvering, and collective teamwork for goal scoring. Yet, it’s important to acknowledge the inevitable randomness in football, where goals can also result from unexpected events such as deflections and own goals. Nonetheless, this study emphasized the significant role played by the build-up events preceding the shot in shaping goal-scoring opportunities.

## Supporting information

S1 File Feature analysis of features from existing literature. (PDF)
