# Supplementary material for: Predicting goal probabilities with improved xG models using event sequences in association football
Source: PLoS One. 2024 Oct 30;19(10):e0312278. doi: 10.1371/journal.pone.0312278 (PMC11524524; doi:10.1371/journal.pone.0312278)
Supplement: S1 File — (PDF) [file pone.0312278.s001.pdf]

S1 Feature analysis of features from existing literature

From the existing literature, it was identified distance to the goal, angle to the goal, duration of the event, the shot played by foot, and opposition pressure on the ball carrier as features that improve the xG accuracy and features that could be extracted from sequences in a time-series manner. However, existing literature has only analyzed these features with shot events. As a novel contribution, this work analyzes these features with events preceding the shot event as well.

For the computation of the distance to goal and angle to goal from event locations, the midpoint of the defending teams' goal coordinates as (120, 40) and two goal post coordinates of the defending team as (120, 36) and (120, 44) in the 120×80 grid was used. The Euclidean distance between the event location and the midpoint of the goal was calculated as the distance to the goal. The angle formed between one goal post, event location, and the other goal post was calculated in degrees as the angle to the goal (Fig S1).

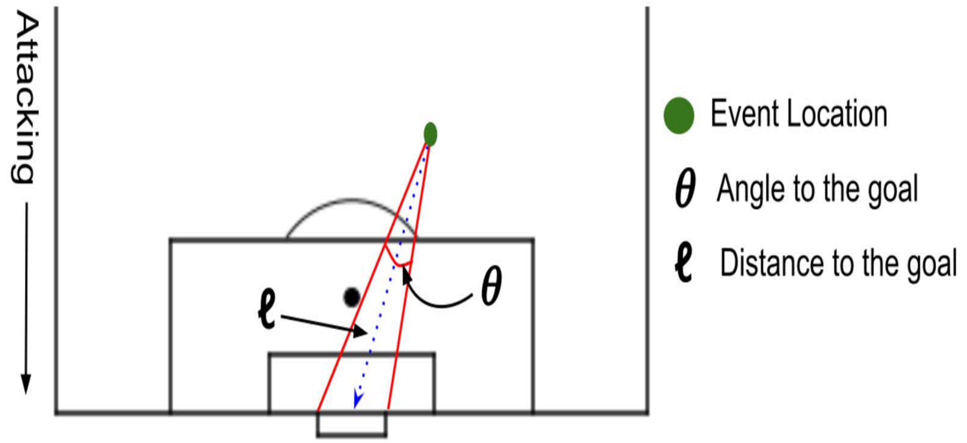

S1 Fig. Distance to the goal and angle to the goal.

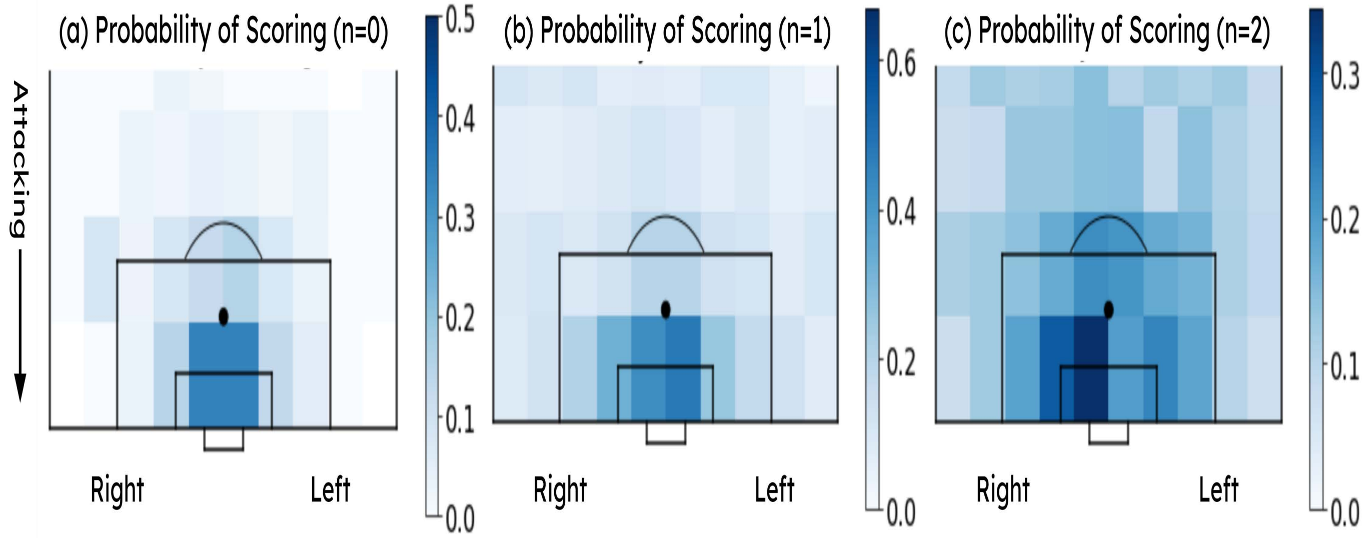

S2 Fig. The probability that a shot will result in a goal against the shot location. Three heatmaps represent the shot locations and the probability of success from that location for events; shot event (a) $n = 0$, last events that occurred before the shot (b) $n = 1$, and second last events that occurred before the shot (c) $n = 2$ event location.

It can be observed from Fig S2 that passes within the box and from sides of the goal within the 18-yard box in preceding events to the shot ($n = 1$, $n = 2$) increase the probability of scoring. Fig S3 and Fig S4 show a relationship between the probability of scoring, the distance to the goal, and the angle to the goal, respectively. The probability of scoring decreases with the distance to the goal for the shot event ($n = 0$). The small bump around 60 could be a result of inconsistency in the data collection or psychological effects of players attempting a shot at goal after the center line (once in opposition territory) if the goalkeeper is not prepared for a save (as the centerline is at 60), but we do not have sufficient information regarding this small artifact. It was noted this artifact does not affect this work's computation. However, the plot becomes more scattered for the preceding events ($n = 1$, $n = 2$). This could be due to scoring from crosses, long passes, counterattacks, and cut-backs. The probability of scoring increases with the angle to the goal of a shot; however, for $n = 2$, a decrease in probability with the increase in angle to the goal could also be observed.

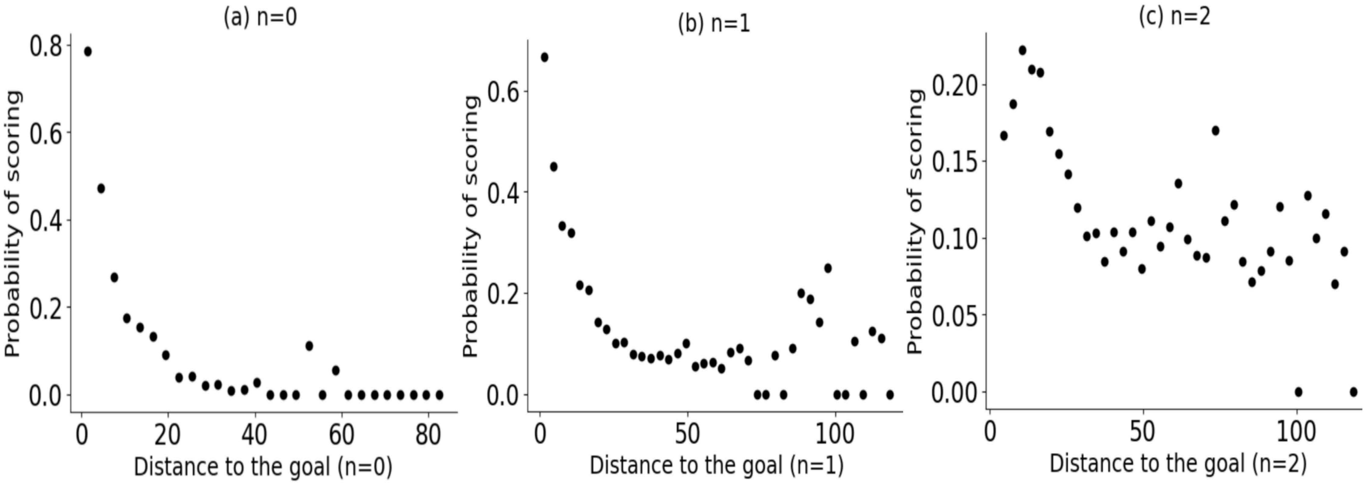

S3 Fig. Probability of scoring against the distance from the event location to goal. (a) $n = 0$ (shot), (b) $n = 1$, (c) $n = 2$. Distances are based on Euclidean distances measured on a 120×80 grid.

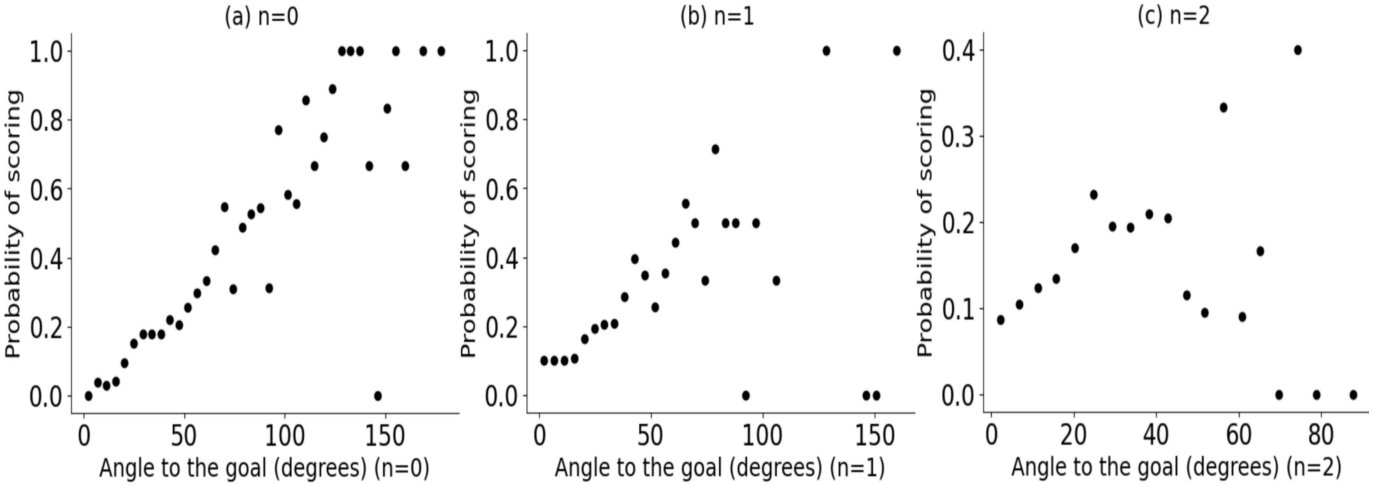

S4 Fig. Probability of scoring against the angle from event location to the goal. (a) n=0 (shot), (b) n=1, (c) n=2. The angle is measured in degrees.

Other common factors, such as the duration of the event (Fig S5), opposition pressure on the ball carrier, and whether the pass/shot was played by foot (Fig S6) were also analyzed for shots and preceding events. The probability of scoring decreases with the duration of the $n = 0$ event (after 1st second) and $n = 1$ event. However, for $n = 1$ probability slightly increases towards the end with higher duration as well. For $n = 2$, the plot is more scattered, and higher scoring probability can be observed for higher event duration values. These high-duration events can occur with longer runs (carries) and when waiting for defenders to commit. The scoring probability significantly drops if the opposition pressures a shot ($n = 0$) (Figure **??**). However, there was a significant increase in the scoring probability when the opposition defense pressures the ball carrier in the $n = 2$ event. This could indicate that the scoring probability increases if the preceding events attract opposition players. Such situations can lead to gaps and open spaces for a successful shot event.

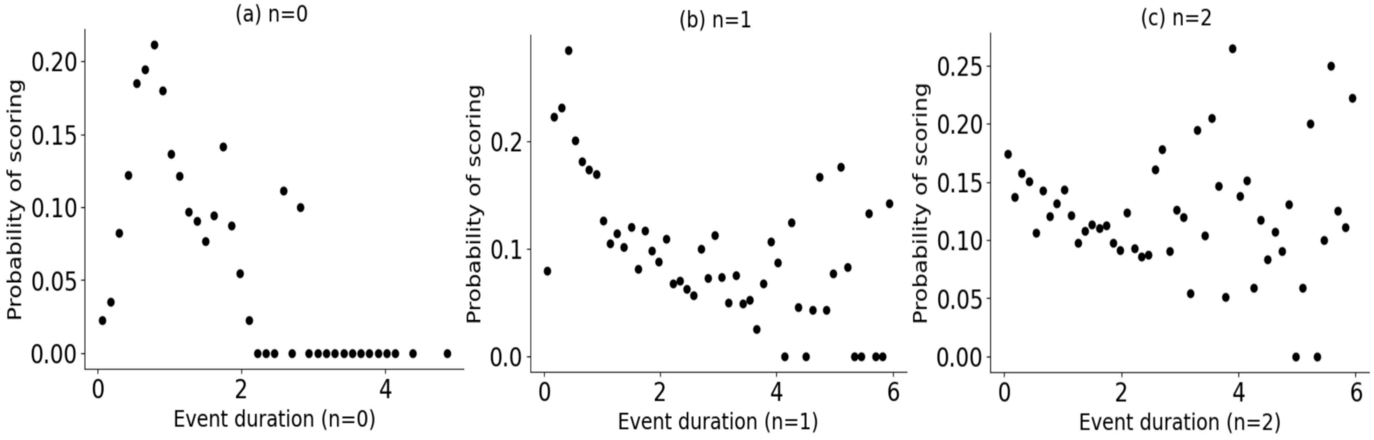

S5 Fig. Probability of scoring against the event duration. (a) $n = 0$ (shot), (b) $n = 1$, (c) $n = 2$. Duration is calculated in seconds.

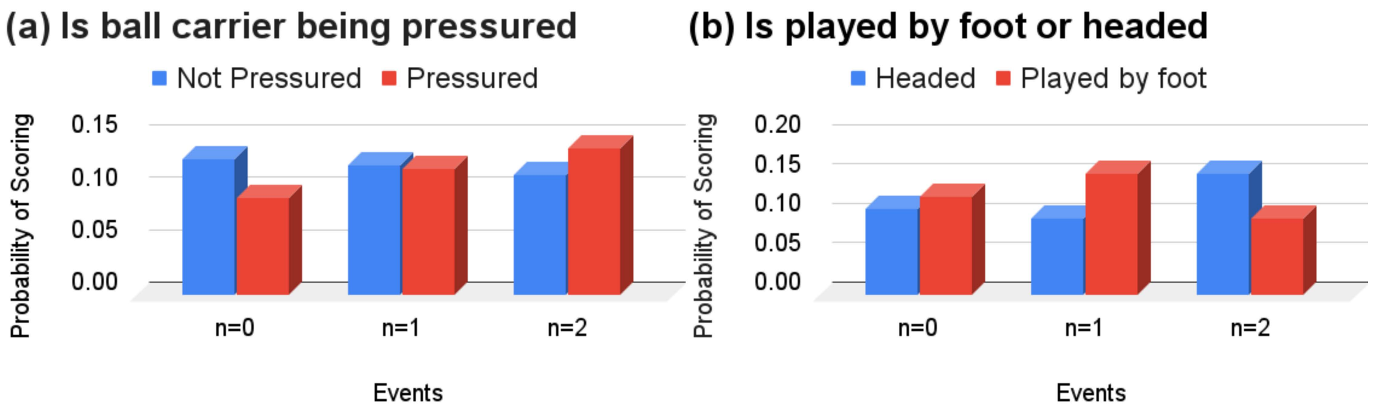

S6 Fig. Probability of scoring against (a) the ball-carrier being pressured by the opposition, (b) played by foot or not.
